# Neural correlates of uncertainty processing: meta-analysis of fMRI studies

**DOI:** 10.3389/fnins.2025.1662272

**Published:** 2025-11-11

**Authors:** Andrey Timashkov, Sarah Anderson, Oksana Zinchenko

**Affiliations:** Centre for Cognition and Decision Making, Institute for Cognitive Neuroscience, HSE University, Moscow, Russia

**Keywords:** uncertainty, decision-making, meta-analysis, fMRI, cascade model, ALE

## Abstract

**Introduction:**

Understanding the neural mechanisms underlying decision-making under uncertainty represents a fundamental challenge in cognitive neuroscience. This meta-analysis aimed to identify the consistent neural correlates of uncertainty processing specifically during decision-making tasks.

**Methods:**

We synthesized findings from 76 fMRI studies (*N* = 4,186 participants). Using the Activation Likelihood Estimation (ALE) method, we performed a voxel-wise meta-analysis of activation foci to identify brain regions consistently activated across studies.

**Results:**

The analysis revealed nine distinct activation clusters, revealing a comprehensive neural network involved in uncertainty processing. Key findings demonstrated predominant activations in the anterior insula (up to 63.7% representation), inferior frontal gyrus (up to 40.7%), and inferior parietal lobule (up to 78.1%). We found a functional specialization between emotional-motivational processes (clusters 1–5) and cognitive processes (clusters 6–9), with notable hemispheric asymmetries. The left anterior insula was more strongly associated with reward evaluation, while the right was involved in learning and cognitive control. Similarly, the right inferior frontal gyrus was linked to impulse control, and the left to motor planning.

**Discussion:**

Our findings extend the current understanding of the neural architecture of decision-making under uncertainty. The comprehensive mapping of neural signatures advances our knowledge of the distinct roles of key brain regions and provides insights into potential clinical applications, particularly for developing interventions for uncertainty-related anxiety. The study highlights important directions for future research in cognitive neuroscience and clinical practice.

## Introduction

Under uncertainty, making decisions is considered as a primary cognitive process which influences how we behave as humans and allow us navigate through unpredictable environment by estimating risks and reward. This capability solely relies on a network of neural activities at certain brain regions, including the dorsolateral prefrontal cortex (dlPFC), anterior insula, and the anterior cingulate cortex (ACC), which are considered essential in integrating sensory information, appraising risk, and modulating emotional responses, crucial for decision-making across different contexts (Morriss et al., 2019) from simple, everyday decisions to complex, high-stake problem despite the lack of complete information (Craig, 2009; Shenhav et al., 2013). These regions of the brain are constantly in the investigation of uncertain processes in neuroimaging research for fields in neuroeconomics, psychology and behavioral neuroscience (Bechara et al., 2000; Kahneman and Tversky, 2013).

Morriss et al. (2019), through a comprehensive coordinate-based meta-analysis, provided foundational insights into the neural circuitry involved in general uncertainty processing. Their findings emphasized the anterior cingulate cortex (ACC) and anterior insula as integrative hubs for cognitive and emotional signals during uncertainty. However, a critical limitation they acknowledged was the broad scope of uncertainty types examined, without isolating those specifically linked to decision-making processes. The present meta-analysis addresses this gap by focusing exclusively on decision-related uncertainty to identify brain regions consistently recruited when individuals evaluate incomplete or ambiguous information in the context of choice. Unlike previous meta-analyses that examined neural correlates of uncertainty across broad contexts (e.g., Morriss et al., 2019), the present study specifically focuses on active decision-making paradigms to identify the core neural substrates of choice under conditions of incomplete information.

Our review contributes to the field with the updated framework based on data from recently published neuroimaging studies by Spohrs et al. (2021) and Savage et al. (2020) illustrating the pivotal roles of the ACC and the anterior insula in tuning emotional responses amid decision-making and Gorka et al. (2015), highlighting the association of subcortical structures like the amygdala in decision making processes that are based on reward under uncertainty, to provide an updated outline of the neural mechanisms activated in the presence of incomplete information and when outcomes are unpredictable (Lissek et al., 2014; Gorka et al., 2016; Visalli et al., 2019). For example, Canessa et al. (2013) discovered that the disparities in decision-making approach when faced with uncertainty are corresponding to contrasting activation patterns in the ACC and the anterior insula. By merging these findings, this paper explores to augment our comprehension of the neural mechanisms of facilitating decision making processed in uncertain situations. This updated composite of neural signatures will propel the field forward by elucidating the distinct roles of the key brain regions activated during decision making processes under uncertainty, underlining future research directions.

To contextualize the expected findings, the cascade model of prefrontal executive function (Koechlin et al., 2003) offers a compelling theoretical framework. This model proposes that the prefrontal cortex supports hierarchical and parallel control processes during decision-making. Medial structures—such as the dorsal anterior cingulate cortex (dACC)—evaluate ongoing strategy reliability, while lateral prefrontal regions, including frontopolar cortex, support the generation and maintenance of alternative strategies. Applying this model to uncertainty may help clarify how the brain dynamically integrates emotional evaluation and cognitive control to optimize decisions in unpredictable environments.

This meta-analysis systematically maps the current landscape of fMRI research on decision-making under uncertainty through Activation Likelihood Estimation (ALE). While advanced methods like meta-analytic connectivity modeling (MACM) or subgroup analyses could offer deeper mechanistic insights, our primary objective is to establish a foundational synthesis of reported neural activations across studies. By focusing on spatial concordance rather than connectivity or subpopulation effects, we provide a comprehensive reference for: (1) identifying consistently reported activation patterns, (2) highlighting methodological and conceptual trends in the literature, and (3) guiding future hypothesis-driven investigations with more complex analytical approaches.

## Materials and methods

### Literature search

**FIGURE 1:**
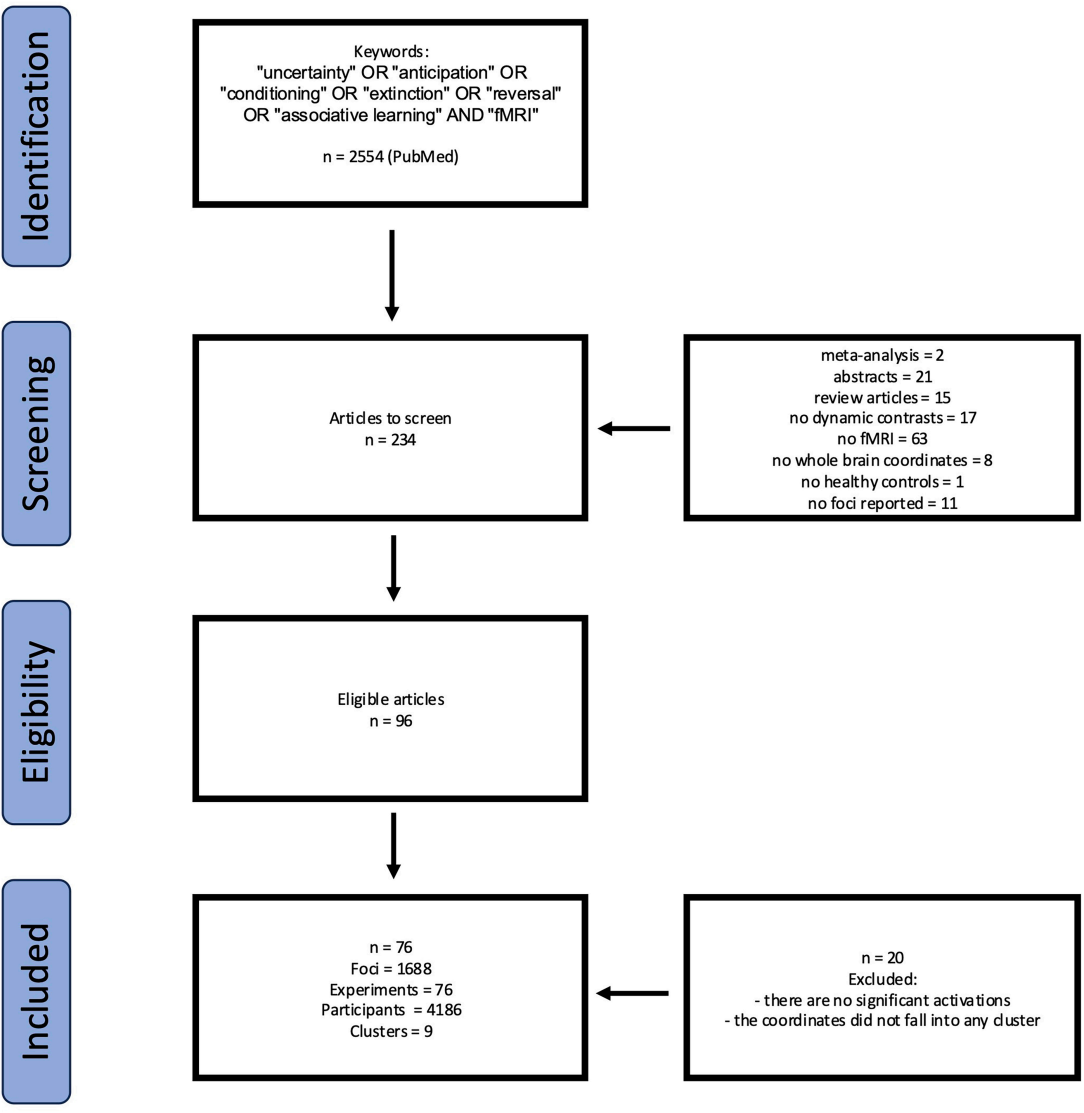
PRISMA flowchart for eligibility of articles.

The initial literature search was conducted in July 2024, with the additional search in January 2025 to account for newly published studies. The literature search was conducted using PubMed database. Articles were selected using the following keywords: “uncertainty,” “anticipation,” “conditioning,” “extinction,” “reversal,” “fMRI” and “associative learning,” years 1995–2024. Initially, 2,554 articles were identified. Following preliminary screening for duplicates and required data (fMRI data and decision-making paradigms under uncertainty), 234 articles were selected for further analysis and subsequent clustering resulting in 76 eligible studies with the data collected on healthy adults, random-effects model. PRISMA chart represents the stages of data collection and selection (Figure 1). Studies were included if they: (1) used fMRI; (2) involved healthy adult participants; (3) employed a task explicitly involving active decision-making under conditions of uncertainty (e.g., risk, ambiguity, unpredictable threat, reversal learning); (4) reported whole-brain coordinates of significant activation contrasts related to uncertainty processing. Studies were excluded if they focused solely on perceptual uncertainty without a decision component or studied exclusively clinical populations.

### ALE approach

This study employed the Activation Likelihood Estimation (ALE) method, which enables voxel-wise analysis of images with random effects. GingerALE 3.0.2 was utilized in the current investigation. The software allows the use of coordinates to create a probabilistic activation map using ALE values. These values provide information about statistical maps of probable activation in relation to the tasks included in the analysis. For data consistency, coordinates presented in Montreal Neurological Institute (MNI) format were converted to Talairach space. Statistical significance was assessed using cluster-level correction for multiple comparisons at *p* = 0.05 and a cluster-forming threshold of *p* < 0.001 (Eickhoff et al., 2012, 2017). The following are the articles included in the final list of publications included in the results of the analysis (Table 1).

**TABLE 1:** Descriptive information of studies and contrasts used in the meta-analysis.

References	*N* (female)	Mean age	Handedness	Sex	Clusters	Task
Wu et al., 2014	52 (29)	50 ± 16.5	Right	M/F	1, 3, 4, 5	Monetary Incentive Delay task (anticipation of gains/losses)
Vytal et al., 2014	20 (7)	28	Right	M/F	1, 2, 5	Threat of shock paradigm (sustained anxiety)
Nitschke et al., 2006	21 (11)	29	Right	M/F	1, 2, 7	Anticipation and exposure to aversive pictures
Canessa et al., 2013	15	25.4 ± 2.45	Right	–	1, 2, 3, 4, 5, 6, 8	Aviation decision making task under uncertainty and financial incentive
Heeren et al., 2017	46	SAD 24.96 ± 6.46, NACP 25.30 ± 5.62	Right	F	1	Virtual game “CYBERBALL” (social inclusion/exclusion)
Savage et al., 2020	94 (64)	21.32 ± 2.28	–	M/F	1, 2, 3, 5	Fear reversal learning task (threat and safety learning)
Gorka et al., 2015	40 (73%)	31.4 ± 12.1	–	M/F	1, 3, 4, 9	Reward anticipation tasks (EEG and fMRI)
Wan et al., 2016	60	30.2 ± 1.5	Right	M	2, 3	Complex problem solving tasks in expertise domain
Mestres-Missé et al., 2017	22 (11)	25.5 ± 3.1	Right	M/F	1.4	Learning task with probabilistic sequences and outcomes
Lim et al., 2020	42 (21)	31.23 ± 10.91	Right	M/F	2, 4, 5	Pain anticipation task with learned cognitive schema
Grandjean et al., 2013	25 (13)	21.8 ± 2.68	Right	M/F	1, 2, 6, 7, 9	Stroop task with item-specific proportion congruent effect
Collins et al., 2017	26 (11)	23	Right	M/F	3, 4, 6, 9	Working memory and reinforcement learning interaction task
Zhang et al., 2013	20 (16)	24.65	Right	M/F	1, 2, 3, 5, 8	Perceptual decision tasks with chosen vs. specified rules
Young and Nusslock, 2016	40 (20)	“PM: 21.19 ± 1.34; NM: 20.86 ± 1.83”	Right	M/F	1, 4	Monetary Incentive Delay task under positive/neutral mood
Votinov et al., 2015	43 (HH/LL)	“LL: 23.6 ± 1.4; HH: 23.04 ± 0.8”	–	–	1, 2, 3	Reversal learning task with genetic polymorphism analysis
Enzi et al., 2012	30 (16)	40.9 ± 11.5	Right	M/F	1, 3, 4, 5, 8	Monetary Incentive Delay task for reward/punishment processing
Bach et al., 2009	20 (10)	27.4 ± 5.8	Right	M/F	6, 7, 8	Task contrasting ambiguity vs. risk in aversive outcomes prediction
Ridderbusch et al., 2021	100 (46)	33.1 ± 10.7	–	M/F	5	Delayed extinction training with reinstatement test
Labrenz et al., 2022	94 (51)	27.04 ± 0.86	–	M/F	4, 5, 7	Interoceptive fear conditioning and safety learning
Ruge and Wolfensteller, 2016	27 (14)	24.6	–	M/F	6, 8, 9	Instructed reversal learning vs. feedback-driven learning
Criaud et al., 2017	29 (19)	20–42	Right	M/F	1, 9	Response inhibition task under different cognitive control models
Stout et al., 2018	25 (10)	34.57 ± 10.13	Right	M/F	6, 7	Contextual threat conditioning with configural/elemental learning
Fleming et al., 2012	26 (10)	28.3	Right	M/F	6	Perceptual decision-making with metacognitive reports
Davis and Hasson, 2018	25 (12)	24.6 ± 3.9	Right	M/F	5	Task examining predictability of object category and location
Bereczkei et al., 2013	27 (14)	23 ± 2.42	Right	M/F	3	Trust game examining Machiavellian strategies
Rodriguez, 2009	14 (8)	21–37	Right	M/F	6, 8	Stimulus outcome association learning with prediction errors
Doornweerd et al., 2018	38 (PAIRS)	49.8 ± 9.8	–	F	1.3	Food reward processing in monozygotic twins
Harnett et al., 2016	23 (10)	19.92 ± 0.59	Right	M/F	8	Temporal fear conditioning paradigm
Stewart et al., 2013	PSU: 18, DSU: 15, CTL: 14	PSU: 24.39, DSU: 24.33, CTL: 24.36	Right	M/F	4	Fear conditioning with inspiratory breathing load
Kokonyei et al., 2019	38 (23)	25.79 ± 4.17	Right	M/F	4, 6	Pain anticipation and perception with fear conditioning paradigm
Zeuner et al., 2016	31 (16)	51.0 ± 13.1	Right	M/F	1, 3, 7	Probabilistic reversal learning task with reward based learning
Liu et al., 2023	30 (16)	21.55 ± 2.32	Right	M/F	1, 2, 3, 5	Sequential risk taking decisions with outcome anticipation
Costumero et al., 2016	45	26.44 ± 5.39	Right	M	3, 4, 5	Gambling task with monetary reward/punishments
Limongi et al., 2016	16 (9)	22.4 ± 5.51	Right	M/F	1, 2	Action update paradigm with temporal estimation
Wang et al., 2020	33	25.1 ± 3.8	Right	M	3, 6, 9	Associative learning task with tactile events
Murty et al., 2016	49 (28)	25 (18–36)	Right	M/F	1, 4, 6, 8	Perceptual surprise task under reward/punishment motivation
Bereczkei et al., 2013	27 (14)	23 ± 2.42	Right	M/F	3, 5	Trust game examining neural operations in social dilemma
Bereczkei et al., 2013	27 (14)	23 ± 2.42	Right	M/F	3, 5	Trust game examining neural operations in social dilemma
Bothe et al., 2013	19 HT, 24 PIA	HT 25.11 ± 3.73, PIA 24.46 ± 2.81	Right	M	4	Monetary Incentive Delay task after acute exercise
Skvortsova et al., 2020	88	–	Right	F	3	Emotional faces, crying baby sounds, and heat pain tasks
Remijnse et al., 2005	27 (19)	32 (22–53)		M/F	1, 3, 8	Reversal learning task with affectively neutral baseline
Vorobyev et al., 2015	215 (17)	17–18	Right	M	1, 4, 5	Computerized driving task examining risk taking behavior
Yin et al., 2018	18 (9)	17–33	–	M/F	1, 3	Classical differential fear conditioning task
van der Heiden et al., 2014	10 (5)	25.3 ± 1.77	Right	M/F	1	Semantic classical conditioning paradigm for BCI communication
Jocham et al., 2009	35	20–32	–	M	5	Probabilistic reversal learning task with genetic analysis
Paul et al., 2015	29 (14)	–	–	M/F	6	Perceptual categorization task with uncertainty responses

## Results

Based on the meta-analysis, which included 1,688 activation foci from 76 experiments with a total of 4,186 participants, key brain structures involved in decision-making processes were identified. The results demonstrate that decision-making engages an extensive neural network encompassing both cortical and subcortical structures in both hemispheres of the brain.

**FIGURE 2:**
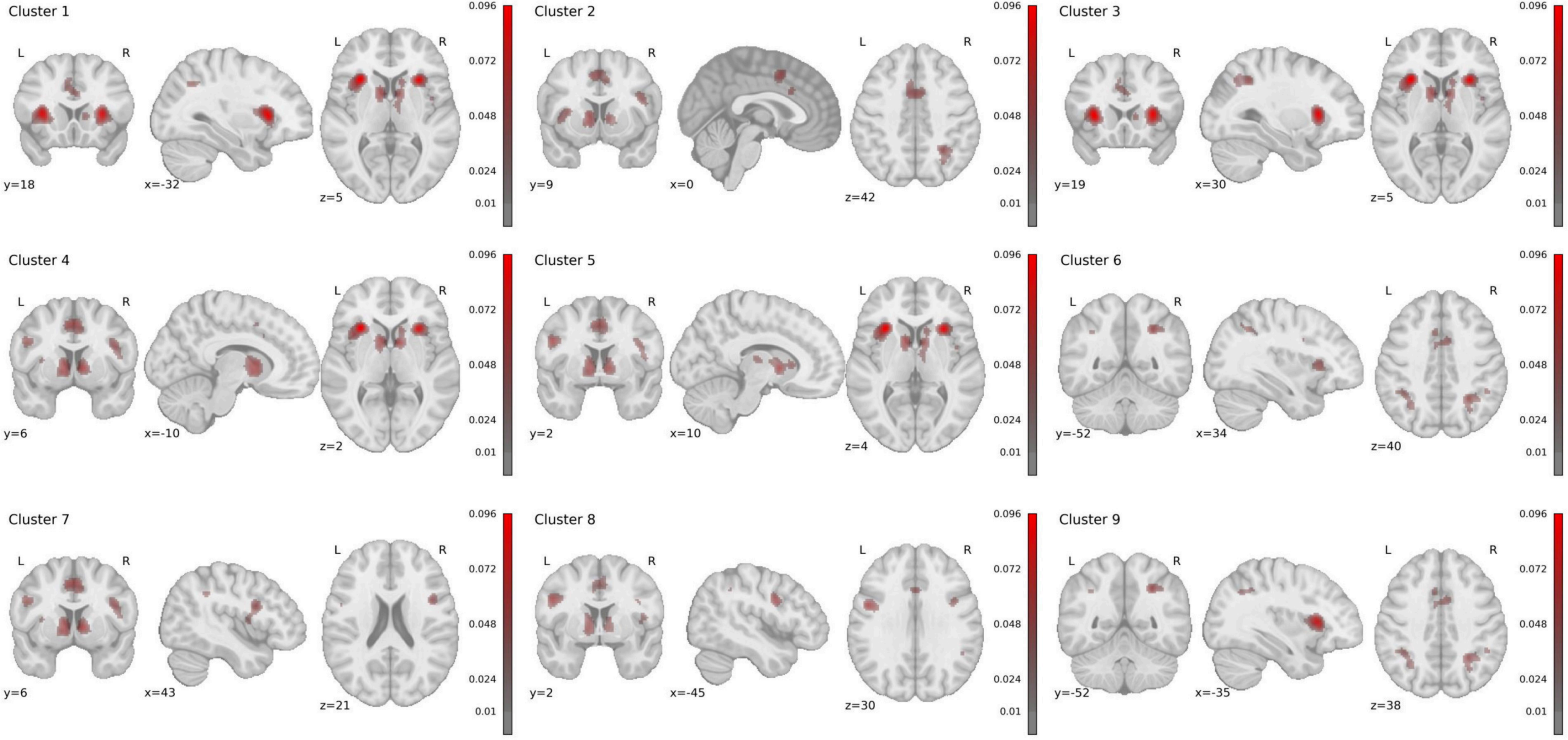
Rendered ALE maps showing significant concordance across studies for uncertainty processing.

We identified 9 statistically significant activation clusters (Table 2). Figure 2 shows a brain activation map. The image was obtained using the Mango program v.4.1.

**TABLE 2:** Concordant brain regions associated with uncertainty processing.

N_^o^_ cluster	Volume (mm^3^)	Peak (coordinates, Z-value)	Hemisphere	Lobe	Gyrus	Tasks contributed
1	4,664	(−30, 22, 4), *Z* = 8.94	Left 100%	Sub-lobar 81.6%, frontal 18.4%	Insula 63.7%, inferior frontal 16.2%, claustrum 16.2%	Monetary Incentive Delay tasks, reward/punishment anticipation, risk assessment under uncertainty. General pattern: anticipation of decisions with emotional valence
2	3,736	(−4, 8, 46), *Z* = 5.47	Left 54.9%, Right 45.1%	Frontal 50.2%, limbic 49.8%	Cingulate 52.9%, medial frontal 27.6%, superior frontal 19.5%	Anxiety stimuli processing, threat anticipation, fear learning. General pattern: assessment of potentially threatening stimuli and uncertainty situations
3	3,152	(30, 20, 8), *Z* = 8.70	Right 100%	Sub-lobar 88.7%, frontal 11.3%	Insula 61.3%, claustrum 22.6%, inferior frontal 12.3%	Reinforcement learning tasks, reversal learning, prediction error processing. General pattern: behavior adaptation based on feedback
4	3,040	(−10, 8, 0), *Z* = 5.50	Left 100%	Sub-lobar 100%	Caudate 64.4%, lentiform nucleus 34.5%, thalamus 1.1%	Decision making under uncertainty, outcome probability assessment, predictable/unpredictable events processing
5	2,872	(10, 6, 2), *Z* = 5.91	Right 100%	Sub-lobar 100%	Caudate 47.9%, thalamus 34.3%, lentiform nucleus 17.9%	Temporal prediction tasks, time interval assessment, temporal event anticipation
6	2,648	(30, −52, 42), *Z* = 5.37	Right 100%	Parietal 100%	Inferior parietal lobule 47.1%, superior parietal lobule 23.5%, supramarginal 14.7%, precuneus 14.7%	Working memory tasks, cognitive control, information updating
7	1,792	(44, 8, 24), *Z* = 4.93	Right 100%	Frontal 68%, sub-lobar 32%	Inferior frontal 36%, insula 32%, precentral 24%, middle frontal 8%	Response inhibition tasks, impulse control, task switching
8	1,568	(−48, 2, 30), *Z* = 5.75	Left 100%	Frontal 100%	Precentral 56%, inferior frontal 40.7%, middle frontal 3.3%	Motor planning tasks, action preparation, decision making with motor component
9	944	(−38, −46, 40), *Z* = 4.11	Left 100%	Parietal 100%	Inferior parietal lobule 78.1%, precuneus 9.4%, angular 6.3%, supramarginal 6.3%	Spatial attention tasks, spatial information processing, sensory information integration

Cluster 1 has a volume of 4,664 mm^3^ and is entirely located in the left hemisphere. The majority (81.6%) consists of subcortical structures, while the remaining 18.4% is in the prefrontal cortex. The anterior insula has the largest representation at 63.7%. Additional activations are present in the inferior frontal gyrus (16.2%) and claustrum (16.2%). The activations correspond to Brodmann areas 13 (59.8%), 47 (10.6%), and 45 (3.9%). Studies in this cluster employed paradigms involving monetary reward and punishment (Monetary Incentive Delay tasks), anticipation of gains and losses, and risk assessment under uncertainty. General pattern: anticipation of decision outcomes with emotional valence.

Cluster 2 has a volume of 3,736 mm^3^ with predominant activation in the left hemisphere (54.9% left, 45.1% right). Activation topography is distributed between the frontal region (50.2%) and limbic system (49.8%). Maximum activity was recorded in the cingulate gyrus (52.9%), medial frontal gyrus (27.6%), and superior frontal gyrus (19.5%). Activations correspond to Brodmann areas 32 (43.6%), 6 (33.5%), and 24 (21.8%). Studies included tasks involving anxiety-provoking stimuli processing, threat anticipation, and fear learning. General pattern: assessment of potentially threatening stimuli and uncertainty situations.

Cluster 3, with a volume of 3,152 mm^3^, is localized in the right hemisphere. It predominantly includes subcortical structures (88.7%) and partially frontal cortex (11.3%). Dominant activity is observed in the anterior insula (61.3%), claustrum (22.6%), and inferior frontal gyrus (12.3%), with inclusion of the lentiform nucleus (2.8%). Activations correspond to Brodmann areas 13 (53.8%), 47 (11.3%), and 45 (2.8%). Studies included reinforcement learning tasks, reversal learning, and prediction error processing. General pattern: behavior adaptation based on feedback. While Clusters 1 and 3 both involve the anterior insula and inferior frontal gyrus, they demonstrate clear hemispheric specialization and distinct anatomical profiles. Cluster 1 (left) shows additional activation in the claustrum, while Cluster 3 (right) includes the lentiform nucleus. Functionally, they are dissociable: Cluster 1 is predominantly associated with motivational anticipation, while Cluster 3 is linked to behavioral adaptation. This pattern suggests complementary rather than identical roles in uncertainty processing.

Cluster 4, with a volume of 3,040 mm^3^, is entirely localized in the left hemisphere within subcortical structures (100%). Primary activations are in the caudate (64.4%), lentiform nucleus (34.5%), and thalamus (1.1%). Detailed analysis revealed activations in the caudate head (34.5%), caudate body (30%), and putamen (12.7%). Studies included decision-making tasks under uncertainty, outcome probability assessment, and processing of predictable and unpredictable events. General pattern: probabilistic information processing.

Cluster 5, with a volume of 2,872 mm^3^, is entirely located in the right hemisphere within subcortical structures (100%). It includes the caudate (47.9%), thalamus (34.3%), and lentiform nucleus (17.9%). Detailed analysis showed activations in the caudate head (26.1%), caudate body (21.8%), and medial dorsal Nucleus (8.9%). Studies included temporal prediction tasks, temporal interval estimation, and temporal event anticipation. General pattern: temporal information processing and prediction. Clusters 4 and 5, while both involving basal ganglia structures, exhibit different anatomical distributions and functional specializations. Cluster 4 (left) is predominantly located in the caudate nucleus, while Cluster 5 (right) shows greater thalamic involvement. Correspondingly, Cluster 4 is associated with probabilistic information processing, whereas Cluster 5 is linked to temporal prediction. These findings indicate distinct subcortical contributions to different dimensions of uncertainty.

Cluster 6, with a volume of 2,648 mm^3^, is localized in the right hemisphere within the parietal region (100%). Primary activations are in the inferior parietal lobule (47.1%), superior parietal lobule (23.5%), and supramarginal gyrus (14.7%). It corresponds to Brodmann areas 40 (54.4%), 7 (36.8%), and 19 (8.8%). Studies included working memory tasks, cognitive control, and information updating. General pattern: maintenance and manipulation of information in working memory.

Cluster 7, with a volume of 1,792 mm^3^, is localized in the right hemisphere. It includes frontal cortex regions (68%) and subcortical structures (32%). Primary activations are in the inferior frontal gyrus (36%), anterior insula (32%), and precentral gyrus (24%). It corresponds to Brodmann areas 9 (44%), 13 (32%), 44 (12%), and 6 (12%). Studies included response inhibition tasks, impulse control, and task switching. General pattern: cognitive control and inhibition.

Cluster 8, with a volume of 1,568 mm^3^, is located in the left hemisphere within the frontal cortex (100%). It includes the precentral Gyrus (56%) and inferior frontal gyrus (40.7%). Activations correspond to Brodmann areas 6 (61.5%) and 9 (38.5%). Studies included motor planning tasks, action preparation, and decision-making with motor components. General pattern: motor planning and execution. Clusters 7 and 8 demonstrate clear hemispheric and anatomical differentiation. Cluster 7 (right) encompasses the inferior frontal gyrus and anterior insula, regions central to inhibitory control. In contrast, Cluster 8 (left) is centered on the precentral gyrus, supporting motor planning functions. This dissociation aligns with established models of lateralized frontal cortex organization, where right-sided regions specialize in control processes and left-sided regions in action implementation.

Cluster 9, with a volume of 944 mm^3^, is localized in the left hemisphere within the parietal region (100%). Dominant activations are in the inferior parietal lobule (78.1%) and precuneus (9.4%). It corresponds to Brodmann areas 40 (81.3%), 39 (12.5%), and 19 (6.3%). Studies included spatial attention tasks, spatial information processing, and sensory information integration. General pattern: spatial processing and attention.

## Discussion

Our meta-analysis of 76 fMRI studies (*N* = 4,186) reveals that decision-making under uncertainty engages a distributed network of nine cortical and subcortical clusters. The most consistent activations occur in the anterior insula (up to 63.7% representation), inferior frontal gyrus (up to 40.7%), and inferior parietal lobule (up to 78.1%), with distinct functional specialization between emotional-motivational processes (clusters 1–5) and cognitive processes (clusters 6–9). These findings align with and extend previous meta-analyses by Morriss et al. (2019) and Feng et al. (2022), who similarly identified anterior insula and anterior cingulate cortex as core hubs for uncertainty processing. Our results provide novel insights into hemispheric specialization, with left anterior insula’s involvement in reward evaluation and right anterior insula’s engagement in learning and cognitive control.

The observed functional dissociation between ventral emotional-motivational networks and dorsal cognitive-control systems aligns with hierarchical models of prefrontal cortex organization–the cascade model of cognitive control (Koechlin et al., 2003; Collins and Koechlin, 2012; Donoso et al., 2014) which posits two coordinated tracks within the prefrontal cortex: a medial track, including the vmPFC and dACC, responsible for evaluating the reliability of ongoing behavioral strategies, and a lateral track, encompassing frontopolar and lateral PFC regions, which facilitates the exploration and implementation of alternative strategies. This model offers a structured account of how the brain manages uncertainty by balancing stability and flexibility in decision-making (Vassena et al., 2014).

While the Cascade Model provides a compelling framework for our findings, other prominent models offer complementary perspectives. For instance, the expected value of control theory (Shenhav et al., 2013) conceptualizes the anterior cingulate cortex as a region computing the value of deploying cognitive control, which aligns with the role of dACC (Cluster 2) in monitoring strategy reliability under uncertainty. Similarly, the Salience Network perspective (Seeley et al., 2007)—comprising the anterior insula and dACC—emphasizes the detection and integration of salient stimuli to guide behavior, a process central to decision-making under uncertainty. Our findings of consistent co-activations in these regions strongly support this network view. The cascade model was chosen for its specific focus on hierarchical control organization, which effectively parses the functional dissociation between medial (evaluative) and lateral (cognitive control) systems observed in our clusters.

Beyond the hierarchical organization within the PFC, which is described by the Cascade Model, our findings can be fruitfully interpreted through the lens of large-scale brain networks. The consistent co-activation of the anterior insula and dorsal anterior cingulate cortex (dACC) (Clusters 1, 2, 3) aligns them with the core of the Salience Network (SN), which is dedicated to detecting and integrating behaviorally relevant stimuli (Seeley et al., 2007). Concurrently, activations in the dorsolateral prefrontal cortex (dlPFC) and inferior parietal lobule (IPL) (Clusters 6, 7, 8, 9) correspond to the Central Executive Network (CEN), which subserves working memory, cognitive control, and goal-directed attention (Vincent et al., 2008; Worthy et al., 2016).

The Triple Network Model (Menon, 2011) posits that the SN plays a crucial role in dynamic switching between the CEN and the Default Mode Network (DMN). We propose that uncertainty represents a potent salient signal that engages the SN. This engagement may facilitate the attenuation of DMN activity (associated with internal mentation) and the subsequent recruitment of the CEN to implement cognitive control strategies necessary for decision-making in unpredictable environments. Thus, the functional dissociation observed in our ALE maps likely reflects the coordinated yet distinct contributions of the SN (for appraisal and alerting) and the CEN (for implementation and control) during uncertainty processing. This network perspective complements the cascade model by describing the broader architectural context in which these medial and lateral PFC systems are embedded.

Medial track engagement is supported by the activation pattern observed in Cluster 2, which includes the dorsal ACC and medial frontal gyrus—regions implicated in conflict detection and appraisal of strategy failure. This mirrors the cascade model’s conceptualization of dACC as a monitor of strategy reliability, particularly during threat anticipation and emotionally salient uncertainty. These findings also align with Morriss et al.’s (2019) finding that ACC integrates cognitive and emotional information during uncertainty.

Lateral track involvement is evident in right-lateralized clusters (e.g., Clusters 3, 6, and 7), encompassing the inferior frontal gyrus and inferior parietal lobule. These areas are involved in response inhibition, working memory, and adaptive control—functions associated with evaluating and switching between alternative strategies, as outlined in the cascade model’s lateral system. The inferior parietal lobule activations (Cluster 6) during working memory tasks further reflect the cascade model’s proposed monitoring of the counterfactual strategy. These mappings not only support the cascade model’s architecture but also underscore how emotional and cognitive circuits are dynamically recruited in parallel to guide behavior under uncertainty.

The bilateral activation patterns reveal complementary processing streams. Left anterior insula’s dominance in reward evaluation (Cluster 1) corresponds to the cascade model’s actor reliability monitoring, while right anterior insula’s involvement in learning (Cluster 3) reflects hypothesis testing for alternative strategies. Similarly, the lateralization in inferior frontal gyrus–right-sided for impulse control (Cluster 7) and left-sided for motor planning (Cluster 8)–suggests parallel uncertainty resolution mechanisms. These findings extend Feng et al.’s (2022) network approach by demonstrating how hemispheric specialization facilitates concurrent strategy evaluation. The prominence of anxiety-related activations in Cluster 2 (cingulate gyrus, medial frontal gyrus) suggests that uncertainty processing abnormalities may stem from disrupted reliability monitoring (Stout et al., 2019). As Morriss et al. (2021) noted, individuals with high intolerance of uncertainty show altered prefrontal activation patterns. Our Cluster 2 findings provide specific neural targets for interventions, particularly for conditions like generalized anxiety disorder where dorsal ACC hyperactivity during threat appraisal may reflect maladaptive hypothesis generation (Buff et al., 2016; Klass et al., 2021; Spohrs et al., 2022).

While our search strategy prioritized terms that allowed for direct comparison with prior meta-analytic work (Morriss et al., 2019), future studies might benefit from incorporating additional keywords such as “cognitive flexibility” and “exploration-exploitation” to capture an even broader spectrum of decision-making under uncertainty. Furthermore, while ALE approach establishes spatial convergence, it cannot assess dynamic interactions central to the cascade model. Future research should employ dynamic causal modeling to test information flow between medial and lateral prefrontal pathways and examine how individual differences in metacognitive ability (Fleming et al., 2012; Jiang et al., 2022) modulate these networks. The convergence between our meta-analytic findings and the cascade model suggests promising avenues for theoretical integration with explicitly test model predictions about dACC-triggered exploration and frontopolar-mediated strategy maintenance using paradigm-driven fMRI designs. Such work would bridge meta-analytic patterns with computational accounts of prefrontal function.

Several limitations of the ALE methodology should be considered. First, ALE relies on reported peak coordinates, which may introduce a bias toward statistically strong foci. Second, the spatial smoothing inherent to the method can inflate the apparent overlap between activations. Third, ALE identifies consistency, not magnitude, of activation across studies. Finally, our approach assumes a degree of homogeneity across studies in design and analysis, which is an inherent challenge in meta-analysis.

It is noteworthy that our meta-analysis did not identify significant convergence in the cerebellum, despite its established role in various cognitive functions. This is likely attributable to the specific nature of the included paradigms. Our analysis focused on value-based and emotional-motivational appraisal under uncertainty, which engages a cortico-striato-thalamic network. In contrast, the cerebellum’s contribution may be more critical for processing uncertainty in contexts that require explicit probabilistic reasoning and the construction of internal models to guide predictions, as demonstrated in tasks of inductive inference (Blackwood et al., 2004). Furthermore, its role is well-established in domains requiring sensorimotor prediction and adaptive behavioral updating (Blakemore et al., 2001; Kobayashi and Hsu, 2017; Johnson et al., 2019). The paradigms in our final sample were not optimized to capture this specific computational demand of the cerebellum. Future research employing paradigms that directly contrast these different forms of uncertainty could help delineate the distinct and shared neural systems involved.

## Conclusion

Current study provides significant insights into the neural mechanisms underlying decision-making under uncertainty. By identifying nine distinct activation clusters, we highlighted the critical roles of the anterior insula, inferior frontal Gyrus, and inferior parietal lobule in uncertainty processing. Our findings confirm previous research regarding the anterior insula’s prominence in reward evaluation and emotional regulation, while also revealing functional specialization within the identified clusters. The observed bilateral activation patterns underscore the complexity of neural networks involved in decision-making, with distinct roles attributed to each hemisphere.

## Data Availability

The original contributions presented in this study are included in this article/supplementary material, further inquiries can be directed to the corresponding author.
